# 24-h Fluid Kinetics and Perception of Sweat Losses Following a 1-h Run in a Temperate Environment

**DOI:** 10.3390/nu6010037

**Published:** 2013-12-19

**Authors:** Eric K. O’Neal, Christina R. Caufield, Jordan B. Lowe, Mary C. Stevenson, Brett A. Davis, Lauren K. Thigpen

**Affiliations:** 1 Department of Health, Physical Education, and Recreation, University of North Alabama, UNA Box 5073, Florence, AL 35632, USA; E-Mails: christinacaufield@gmail.com (C.R.C.); jlowe@una.edu (J.B.L.); mcstevenson@una.edu (M.C.S.); lkthigpen24@gmail.com (L.K.T.); 2 Department of Health and Human Performance, Middle Tennessee State University, Murfreesboro, TN 37132, USA; E-Mail: bad4e@mtmail.mstu.edu

**Keywords:** beverage choice, urine specific gravity, hydration, running

## Abstract

This study examined 24-h post-run hydration status and sweat loss estimation accuracy in college age runners (men = 12, women = 8) after completing a 1-h self-paced outdoor run (wet bulb globe temperature = 19.9 ± 3.0 °C). Sweat losses (1353 ± 422 mL; 1.9% ± 0.5% of body mass) were significantly greater (*p* < 0.001) than perceived losses (686 ± 586 mL). Cumulative fluid consumption equaled 3876 ± 1133 mL (218 ± 178 mL during) with 37% of fluid ingested lost through urine voids (1450 ± 678 mL). Fluid balance based on intake and urine production equaled +554 ± 669 mL at 12 h and +1186 ± 735 mL at 24 h. Most runners reported euhydrated (pre-run urine specific gravity (USG) = 1.018 ± 0.008) with no changes (*p* = 0.33) at hours 12 or 24 when both genders were included. However, USG was higher (*p* = 0.004) at 12 h post-run for men (1.025 ± 0.0070 *vs.* 1.014 ± 0.007), who consumed 171% ± 40% of sweat losses at 12 h *vs.* 268% ± 88% for women. Most runners do not need intervention concerning between bout hydration needs in temperate environments. However, repeated USG measurements were able to identify runners who greatly under or over consumed fluid during recovery. Practitioners can use multiple USG assessments as cheap method to detect runners who need to modify their hydration strategies and should promote assessment of sweat losses by change in body mass, as runners had poor perception of sweat losses.

## 1. Introduction

Fluid replacement guidelines for athletes during training and competition continue to evolve. Former more aggressive recommendations of blanketing intake volumes prescribed [1] have been replaced with more conservative fluid intake strategies that are more individualized and based on each athlete’s sweat losses [2,3] and even more simple recommendations of merely drinking to thirst have been advocated [4] in the running community and supported by evidence from Dion, *et al*. [5]. While voluntary fluid intake of endurance athletes during training have been investigated in depth [6,7,8], fewer studies have examined the effects of ad libitum fluid consumption and its effect on hydration status following training bouts. With the considerations that most runners’ training runs will be of relatively short duration (<90 min) and fluid consumption is likely to be considerably less than sweat production [8,9], we contend that between bout fluid replacement may be equally if not more important than fluid intake during exercise for runners under most conditions.

Armstrong, *et al*. [10] provide data concerning day-to-day fluid intake and hydration status indicator references for free living men, and several investigations have documented hydration status changes over multiple days in elite Kenyan runners [11,12,13]. In regards to recreational runners, we are aware of only one study that has measured extended fluid turnover. This study included only five men and no women in a cool environment (mean regional high dry temperature = 14 °C) with no control for running volume [14]. Post-run *ad libitum* fluid intake has also been measured over short durations (≤6 h) following exercise [15,16]. While each of these investigations has expanded the base of knowledge concerning between bout rehydration, methodological differences such as short duration of observations, no information concerning exercise-induced sweat losses, little or no physical activity of participants, and an inability to compare differences between genders in similar scenarios limit the interpretation of these investigations to the large and growing body of recreational runners. Therefore, the primary purpose of this investigation was to describe fluid kinetics by direct measurements of fluid intake and urine output and hydration status via urine specific gravity (USG) in endurance-trained male and female runners for ~24 h following a 1-h self-paced run. Additionally, as the majority of fluid intake guidelines [2,3] are based on the premise that quantifying sweat losses is an integral component of determining fluid intake, we examined runners’ ability to estimate their run sweat losses post-run. We hypothesized that female runners would rehydrate more completely than male runners, and that both genders would vastly underestimate sweat losses.

## 2. Methods

### 2.1. Participants

The investigators recruited trained runners of varying ability. To be included, participants had to report being capable of comfortably completing a minimum of 1 h of continuous running on a challenging outdoor course and pass a basic health screening. College age (20 ± 2 years) runners (body mass; men = 73.2 ± 6.3 kg, women = 63.0 ± 5.5 kg) participated in this study. Our sample included current and recent members from men’s (*n* = 7) and women’s (*n* = 3) NCAA Division II cross-country teams and men (*n* = 5) and women (*n* = 5) from a collegiate recreational running club. Participants were recruited by word of mouth and at team or club meetings. Subjects reported averaging 30 ± 17 miles per week across 5 ± 2 running sessions per week during the previous year. All subjects completed a PAR-Q and health questionnaire with no contraindication for physical activity and normal blood pressure was confirmed prior to physical activity. All procedures in the investigation were approved by the local Review Board of Human Subjects Committee. Subjects gave written informed consent prior to data collection.

### 2.2. Procedures

Subjects were asked to report to the laboratory in the months of March and April, well rested and prepared for a late afternoon (4:00–6:00 pm depending on work/school schedule) run. Participants were also asked to bring a spare set of running shorts, t-shirt, and undergarments to be worn during pre and post weighing. Following consent form and health screening procedures, subjects were asked to provide a pre-run urine sample. The investigators gave no hydration recommendations prior to testing as to ensure a spontaneous urine sample could be collected. Urine specific gravity (USG) was assessed with a manual refractometer (SUR-NE 300, Atago, Tokyo, Japan) in duplicate by two investigators. Subjects’ weights were recorded to the nearest 0.1 kg on an electronic scale (BWB-800, Tanita Co., Tokyo, Japan) with subjects wearing only shorts, t-shirt and undergarments. The same dry clothing was worn for post-run weight measurements. All running attire including shoes and undergarments and a towel subjects used to dry themselves off with after their run were weighed (KD-200-210, Tanita Co., Tokyo, Japan) to the nearest 2 g before and after exercise to determine the volume of sweat loss retained on the skin and in the runners’ clothing.

Runners were fitted with a heart rate monitor (Team2 System, Polar Electro, Kempele, Finland) that recorded HR averages over 5 s intervals continuously and were then escorted to a 5 km road route. Subjects were allowed to warm-up briefly, if they desired, and then ran loops around the course at a self-selected pace for ~1 h. If the runner’s pace was too slow or fast to allow for a finishing time of two laps to be between 55 and 65 min on the 5-km course, subject ran laps on an alternate 1-km until 60 min of running were completed. Chilled (3–4 °C) 250 mL bottles of water with spill-proof nozzles were provided to participants at the starting line and halfway point of the 5 km course (*i.e.*, all runners had the opportunity to consume at least 705 mL of water during their run). Based on a previous investigation from our laboratory using the same running course and duration of running in hotter weather [9], we did not expect runners to desire to exceed 705 mL of fluid consumption. The bottles were weighed before and after runs to determine fluid volume consumed. Runners also were instructed not to spit or spray water on their face while running. Wet bulb globe temperature was recorded every 10 min (TH-8, Physitemp Instruments Inc., Clifton, NJ, USA).

Upon returning to the laboratory, subjects entered a privacy room and were presented with two 3 L pitchers of water and a large stack of race aid station style paper cups. Runners were asked to use the pitchers of water to fill the cups with a volume of fluid representing the amount of sweat they believed to have lost during their run. Upon completion of their estimation, the cups were placed on a digital scale and subjects were allowed to see the weight of the cups and change their estimation if desired. Runners then changed back into their dry shorts, t-shirt and undergarments to have their post-run weight recorded. Sweat loss was calculated as difference in pre- to post-run body mass with consideration for urine voids made after the initial weigh assessment and water consumed during the run if applicable.

Investigators placed a variety of sodas, diet sodas, sport beverages, non-caloric sport beverages, juices, and water in ice-filled coolers prior to the run. All bottles were previously weighed to the nearest 2 g and marked so changes in bottle mass could be used to determine volume of fluid consumed when runners returned their bottles. Before leaving, runners were asked to take as many bottles of beverages as they believed they would consume before reporting back to the laboratory the following morning. Subjects were instructed that they could drink as little or as much from each bottle as desired and to not be conservative when determining how many bottles they would take home. Runners were instructed to place any bottles they drank from within 1 h of the time they were given the fluids in a labeled bag and place all other bottles in a separate bag so 1 h post-run fluid consumption could be assessed. Runners were only allowed to drink from the bottles taken from the laboratory. Coffee, tea, alcohol and milk consumption were not allowed as investigators could not accurately account for their consumption. Dietary intake was recorded by participants but not controlled. No outside exercise was allowed during data collection. Runners were also provided with overnight urine collection containers so void volumes could be calculated.

Subjects returned to the laboratory between 7:00 and 9:00 am and provided an additional urine sample that was assessed for USG and weighed to be calculated into the total urine losses for the first 12 h post-run. Subjects completed a written form stating they only drank fluids provided to them, collected all urine voids, and did not engage in any strenuous physical activity since leaving the laboratory for the 0–12 h and 12–24 h sessions. One female participant reported forgetting to collect a urine void. Her data was excluded from analysis when appropriate. Subjects chose a new set of chilled fluid bottles to take with them and were given a new urine collection container to use until they reported back in the afternoon for a final weight, USG, urine volume, and fluid consumption volume assessment.

### 2.3. Statistical Analyses

All data are displayed as mean ± SD. Independent sample *t*-tests were used to determine if differences existed between men *vs.* women and collegiate cross-country runners *vs.* club runners when applicable. Paired-sample *t*-tests were used to compare sweat loss estimations to actual sweat losses. Repeated-measures ANOVA and Bonferonni post hoc test (when applicable) were used to examine differences in pre-run, morning, and evening USG for all runners.

## 3. Results

The average WBGT, dry temperature and relative humidity for the runs were 19.9 ± 3.0 °C, 23.6 ± 3.9 °C, 54% ± 14% respectively. Mean distance equaled 11.7 ± 1.8 km, with an average run time of 59.8 ± 3.4 min at a pace of 5.26 ± 0.91 min/km (HR = 180 ± 9 bpm). Cross country team members ran further (*p* = 0.006) (12.7 ± 1.8 km) and at a faster pace (*p* = 0.001) (4.65 ± 0.63 min/km) than the running club members (10.6 ± 1.0 km; 5.86 ± 0.74 min/km). Sweat losses equaled 1.9% ± 0.5% of body mass (1.6% ± 0.6% of body mass with consideration for run fluid intake) and did not differ (*p* = 0.64) between running club members (1307 ± 373 mL) and cross country runners (1398 ± 483 mL), but did differ (*p* < 0.001) between genders (men = 1584 ± 392 mL, 22 ± 5 mL/kg body mass; women = 1006 ± 121 mL, 16 ± 2 mL/kg body mass).

**Figure 1:**
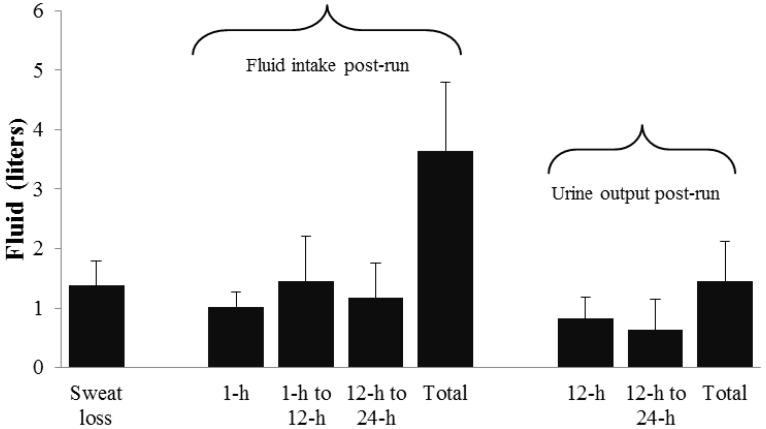
Fluid kinetics by time period (*n* = 19).

Men (209 ± 156 mL) consumed no difference (*p* = 0.55) in water during their run compared to women (261 ± 220 mL). Despite significant differences in sweat loss volumes, there were no differences (*p* = 0.33) in total volume of fluid consumed between men (3800 ± 1250 mL; 52 ± 14 mL/kg body mass) and women (3277 ± 954 mL; 53 ± 14 mL/kg body mass). Fluid volume ranged from 2.7 to 6.4 L for men and 2.0 to 4.5 L for women. There were no differences between genders in percentage of fluid consumption by beverage type (*i.e.*, sodas, diet sodas, juices, caloric sport beverages, non-caloric sport beverages, or water) between men and women with water (34% ± 26%) and caloric sport beverages (34% ± 19%) dominating fluid consumption. Although not statistically different (*p* = 0.34), women produced 1648 ± 776 mL of urine compared to 1334 ± 620 mL for men, contributing to the higher percentage of fluid retention in men than women (65% ± 10% *vs.* 51% ± 20%; *p* = 0.04). Fluid consumption and urine production by time periods following the run are displayed in Figure 1.

**Figure 2:**
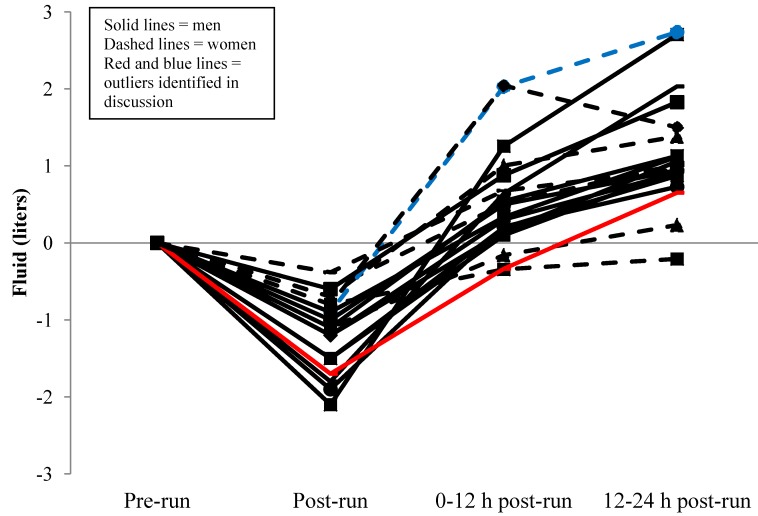
Individual fluid turnover based on sweat losses, fluid intake, and urine output (*n* = 19).

**Figure 3:**
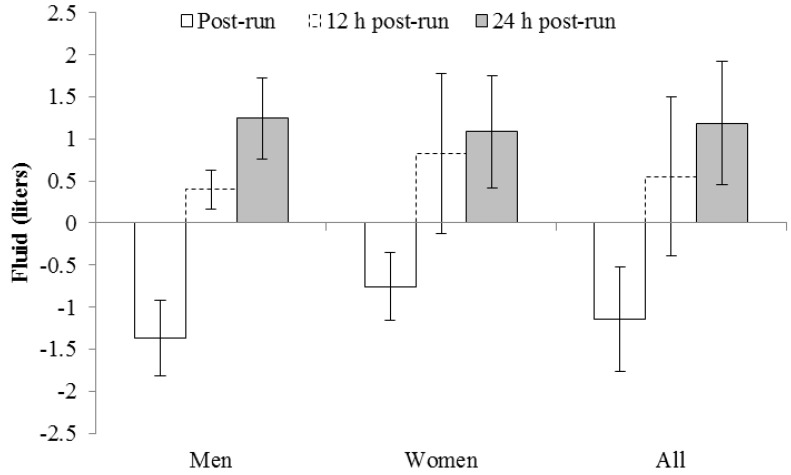
Group fluid turnover based on sweat losses, fluid intake, and urine output (*n* =19).

Based on fluid consumption and urine production, one male and two female runners failed to return to pre-run body water status at 12 h, with one of the female runners still failing to return to pre-run conditions at the 24 h mark (Figure 2). Fluid turnover by group means and combining both genders is displayed in Figure 3.

**Figure 4:**
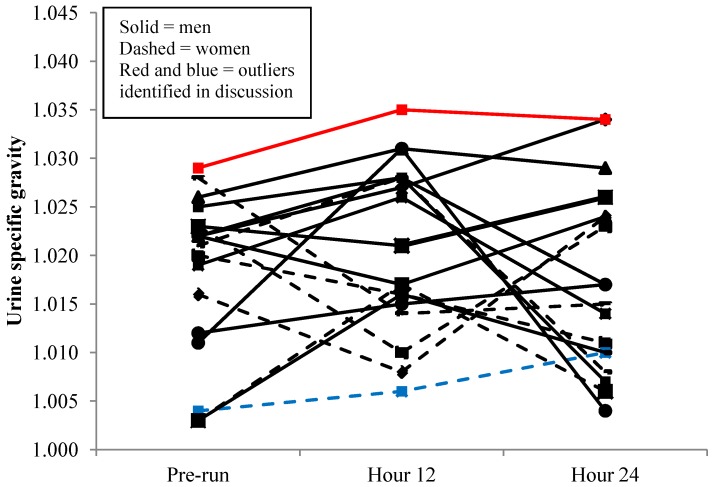
Individual changes in urine specific gravity (*n* = 19 *). * 1 male was unable to provide a pre-run urine sample.

Urine specific gravity values are displayed by individual in Figure 4 and as group means in Table 1. The only statistical difference between genders occurred the morning after the run, and there were no differences among any collection periods when data from both genders were combined (Table 1).

**Figure 5:**
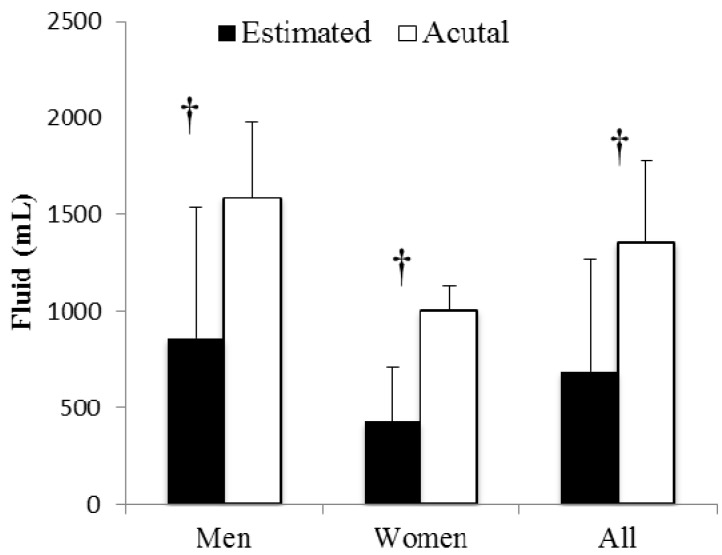
Sweat loss estimation *vs.* actual sweat loss. **†** Indicates sweat loss estimation significantly less than actual sweat loss (*p* < 0.001).

There were no differences in sweat loss estimation accuracy based on percentage when comparisons were made between genders (*p* = 0.56) or between cross country *vs.* club runners (*p* = 0.38). Runners greatly underestimated (*p* < 0.001) their sweat losses based on volume (Figure 5) equaling underestimation of 50% ± 38% (95% CI = 32%–68% of actual sweat loss). Only three of the 20 runners estimated their sweat loss was greater than their actual sweat losses. Sweat retained in clothing items and on the skin after the run equaled 148 ± 105 mL or 11% ± 6% of total sweat losses.

## 4. Discussion

This cross sectional design study was conducted to provide insight to alterations in fluid kinetics and utility of urine specific gravity to detect hypohydration in trained but non-elite college age runners before and after a 1-h outdoor run and hours 0–1, 1–12, and 12–24 post-run. Accuracy in perception of sweat losses was also assessed. The time phases for data collection were chosen based on the consideration that runners often train in the morning or evening, and that running sessions likely take place either 12 or 24 h apart. Under such scenarios, a short period between sessions (e.g., an evening run followed by next morning run) might not allow sufficient time for thirst to result in fluid intake great enough to overcome sweat and subsequent urine losses and return runners to pre-run hydration status. We also postulated that when runs were separated by 24 h, a decrease in thirst stimulus away from exercise stimulus might result in hypohydration. Based on *ad libitum* fluid intake through liquid sources only and urine output in moderate environmental conditions most runners will return to or exceed previous pre-run hydration levels at 12 and 24 h of recovery from a 60 min run.

Several interesting trends are revealed when Figure 1, Figure 2, Figure 3 and Figure 4 are critiqued globally. The first is that fluid intake in the first 12 h post-run is much greater; particularly in the first hour, than hours 12–24 during recovery (Figure 1). The overall percentage of fluid consumption is even more telling since the participants were likely sleeping during 6–8 h of the first 12 h of recovery. Similar trends of large immediate bolus fluid consumption post-run have been previously documented [8,9] and highlight the importance of having fluids available immediately after training. While the physiological mechanisms of the thirst stimulus are complex [17], the large shift in total body water post-run and the fact that most runners consumed dinner and breakfast *vs.* a single lunch meal likely also explains the discrepancies in fluid intake patterns. Initial thirst fluid deficit is mostly satiated within 1 h post-exercise as fluid intake clearly dissipates and is relatively equal when the remaining 23 h are divided, but is adequate to return most runners to the status prior to pre-exercise status.

The second revealing trend was that fluid intake variance differed drastically between genders with men replacing 171% ± 40% of sweat losses *vs.* female runners replacing 268% ± 88% 12 h post-run, and that USG reflected these trends. Individual (Figure 2) and mean (Figure 3) data reveal a tight pattern of slightly positive fluid gain by the previous morning for men but greater relative fluid consumption and retention, even with two negative outliers, in the first 12 h for women. The greater relative increase in fluid retention for women at 12 h is further increased given male runners averaged 10 kg greater body mass. This trend appears to diminish at 24 h though with both genders returning to their respective pre-run USG levels (Table 1 and Figure 4).

**Table 1:** Urine specific gravity kinetics (mean ± SD).

	Men (*n* = 11) *	Women (*n* = 8)	All runners (*n* = 19)
Pre-run	1.020 ± 0.008	1.016 ± 0.010	1.018 ± 0.008
12 h post-run	1.025 ± 0.007	1.014 ± 0.007 ^†^	1.021 ± 0.009
24 h post-run	1.020 ± 0.010	1.014 ± 0.007	1.017 ± 0.010

^†^ Significant difference between male and females runners (*p* = 0.004); * 1 male participant was unable to provide a pre-run sample and was excluded from analysis.

When spontaneous pre-exercise urine samples are acquired, multiple studies have found higher levels of USG in male runners [18], recreational exercisers [19], and team sport athletes [20,21,22,23,24] than their female counterparts. With consideration as to why spontaneous USG is commonly found to be higher in men than women, our data reveals that women consumed 3.3 mL of fluid for every mL of sweat loss compared to 2.4 mL in male runners. While not statistically different, women averaged over 300 mL more urine production despite 500 mL less fluid intake, likely contributing to the lower morning USG levels for women. We cannot explain why women appear to rehydrate more aggressively than men, but hypothesize psychological and social factors concerning the importance of hydration for health, not physiological factors relating to thirst, are attributable for the discrepancies. Baker, *et al*. [25] found older women consume more fluid than men during cycling exercise. Female runners appear to consume fluid at a greater relative rate than men during marathons [26], and even female hikers have been shown to be at a much greater risk of hyponatremia due to overconsumption of fluid compared to male counterparts [27].

In addition to possible psychological differences between genders in regards to fluid consumption, hormonal responses are an obvious factor that may explain some of the discrepancies in fluid consumption. Menstrual cycle phase was not assessed in our investigation. However, although plasma osmolality shifts to a lower level during the luteal phase of the menstrual cycle and during oral contraceptive administration Stachenfeld, Silva, Keefe, Kokoszka and Nadel [16] did not find a difference in thirst ratings, urine output, or *ad libitum* fluid intake during 150 min of light intensity exercise (sweat loss = ~2.3% of body mass) and 3 h of recovery in young women. This is the only study we are aware of in which prolonged fluid kinetics have been compared between male and female runners in a natural environment. Similar future investigations with larger cohorts could confirm if the trends found in this study explain the discrepancies in hydration status of free living men and women.

On separate occasions, Casa *et al*. [28] had male and female subjects run for 1 h after 3:00 pm in the summer (WBGT = ~26 °C) and drink *ad libitum* until a 12-km trail run the following afternoon. Fluid intake and urine output were not measured directly, but assessed mean changes in morning body mass averages from a 3-day baseline equaled −0.79% ± 0.95% and −0.89% ± 1.19%. While the mean changes in body mass seem insignificant, it must be taken into consideration that first morning assessment should represent the athlete’s lightest body mass of the day since two more meals and fluid were consumed post measurement. Additionally, the standard deviations denoted that at least some of the runners began racing at <2% baseline body mass. The impetus for inclusion of individual data is due to our philosophy that most athletes hydrate adequately on their own, and only runners or athletes who train in a state of habitual hypohydration are in need of significant intervention. Day-to-day change in body mass is a good indicator of hydration status, but it is plausible that some athletes train in a chronic state of hypohydration. USG trended similarly with fluid ingestion and sweat/urinary losses. Several repeated USG assessments may be a relatively cheap and effective way to determine habitually hypohydrated athletes.

An excellent example of identification of such an athlete was depicted in this study. The male runner with the highest USG at every time point (pre-run = 1.029, 12 h = 1.034, and 24 h = 1.035) in Figure 2 (denoted by red line) was also the only male runner in a negative fluid balance at 12 h and experienced the greatest fluid deficit of all male runners at 24 h (Figure 4; denoted by red line). This participant replaced only 102% and 160% of run fluid losses at 12 and 24 h, drank nothing during his run, and had an 81.3% fluid retention rate. He was one of the faster runners and reported averaging 55 miles of running per week. Conversely, the slowest female runner vastly over consumed fluid in comparison to sweat losses (blue dashed line in Figure 2) and exhibited one of the lowest trends of USG across collections (blue dashed line Figure 4). While harmless under the conditions of this study, similar hyperhydration behavior transferred to an extended duration run, such as a marathon, could be catastrophic and is a textbook example of characteristics (slow, female runner who consumes copious fluid) exhibited in race day runners with hyponatremia.

Sweat loss perception was of particular interest to the investigators as a relatively accurate estimation of sweat losses is required for effective implementation of American College of Sports Medicine (ACSM) [3] and National Athletic Trainers’ Association (NATA) [2] fluid intake guidelines both during and between training bouts. Having knowledge of sweat losses and implementing appropriate fluid replacement could potentially increase training quality of the hypohydrated runner and ensure excess fluid consumption would not occur during a long run for the hyperhydrated runner described above. Sweat loss underestimations in this study (Figure 5) were similar to those found by Passe *et al*. [8] when a pencil and paper estimation were used (underestimation = 43%) and support underestimation consistency in the hotter environment runs (underestimation = 50%) [9] using the same physical assessment approach of filling race aid station cups with water to represent sweat losses. In support of O’Neal *et al*. [9] no differences in estimation accuracy were exhibited between genders. Sweat on clothing and skin were half of that found in O’Neal *et al*. [9] but did not alter estimation accuracy. Age and running experience also do not appear to affect sweat loss perception as the runners in this study were 20 years younger than the runners in the previous studies [8,9].

Several factors should be considered when interpreting this data. The first is that fluid consumption was potentially decreased because only water and no sport beverages were available for consumption during the run, and several beverages such as milk and tea, which are commonly consumed in the region, were not made available to participants during recovery. Conversely, fluid consumption may have been artificially increased due to the Hawthorne effect and since participants had such free and readily available access to different beverage types. To increase the ecological validity of pre-run USG no instructions were given concerning how to hydrate before reporting to the laboratory. A consequence of this lack of uniformity in preparatory drinking may have resulted in some participants consuming a bolus of fluid shortly to reporting the laboratory and subsequently resulting in a lower USG level. It should also be noted that USG may be more susceptible to incorrectly classifying hydration status in comparison to other measures such as plasma osmolality. Respiratory tract and fecal losses of fluids were not measured, and food water was not considered in changes of body fluid status. Participants recorded food intake in a journal, but the investigators determined the vast inter-individual differences in food quantity, type, timing, and likely inaccuracies in reported serving size made interpretation of food intake on fluid kinetics data difficult.

## 5. Conclusions

The primary findings of this study are that most trained runners will; (A) return to their pre-run hydration status within 12 and 24 h simply drinking to thirst; (B) female runners tend to rehydrate to a greater relative extent than male runners in the first 12 h post-run, but men catch up by 24 h; and that (C) NATA and ACSM [2,3] guidelines which are heavily based on knowing sweat losses do not appear to be relevant to this population as most runners are unaware of their sweat loss volumes. For runners or nutritionists assisting runners in developing hydration strategies, repeated USG assessments can be used to detect if intervention is necessary. Promoting determination of sweat losses by measuring changes in body mass pre- and post-run can then be used to accurately determine fluid needs.
